# Polysialic Acid Neural Cell Adhesion Molecule (PSA-NCAM) is an adverse prognosis factor in glioblastoma, and regulates olig2 expression in glioma cell lines

**DOI:** 10.1186/1471-2407-10-91

**Published:** 2010-03-10

**Authors:** Marie-Claude Amoureux, Béma Coulibaly, Olivier Chinot, Anderson Loundou, Philippe Metellus, Geneviève Rougon, Dominique Figarella-Branger

**Affiliations:** 1 Université de la Méditerranée CNRS UMR6216, Institut de Biologie du Développement de Marseille Luminy, Marseille, France; 2 Abcys SA, 5, rue Pierre Chausson, Paris, France; 3 Université de la Méditerranée, CRO2 UMR911, Marseille, France; 4 Service d'Anatomie Pathologique et de Neuropathologie, Hôpital de la Timone, Marseille, France; 5 Unité de Neurooncologie, Hôpital de la Timone, Marseille, France; 6 Unité d'Aide méthodologique à la Recherche Clinique et Epidémiologique, DRRC, AP-HM, Marseille, France; 7 Service de Neurochirurgie, Hôpital de la Timone, Marseille, France

## Abstract

**Background:**

Glioblastoma multiforme (GBM) is the most aggressive and frequent brain tumor, albeit without cure. Although patient survival is limited to one year on average, significant variability in outcome is observed. The assessment of biomarkers is needed to gain better knowledge of this type of tumor, help prognosis, design and evaluate therapies. The neurodevelopmental polysialic acid neural cell adhesion molecule (PSA-NCAM) protein is overexpressed in various cancers. Here, we studied its expression in GBM and evaluated its prognosis value for overall survival (OS) and disease free survival (DFS).

**Methods:**

We set up a specific and sensitive enzyme linked immunosorbent assay (ELISA) test for PSA-NCAM quantification, which correlated well with PSA-NCAM semi quantitative analysis by immunohistochemistry, and thus provides an accurate quantitative measurement of PSA-NCAM content for the 56 GBM biopsies analyzed. For statistics, the Spearman correlation coefficient was used to evaluate the consistency between the immunohistochemistry and ELISA data. Patients' survival was estimated by using the Kaplan-Meier method, and curves were compared using the log-rank test. On multivariate analysis, the effect of potential risk factors on the DFS and OS were evaluated using the cox regression proportional hazard models. The threshold for statistical significance was p = 0.05.

**Results:**

We showed that PSA-NCAM was expressed by approximately two thirds of the GBM at variable levels. On univariate analysis, PSA-NCAM content was an adverse prognosis factor for both OS (p = 0.04) and DFS (p = 0.0017). On multivariate analysis, PSA-NCAM expression was an independent negative predictor of OS (p = 0.046) and DFS (p = 0.007). Furthermore, in glioma cell lines, PSA-NCAM level expression was correlated to the one of olig2, a transcription factor required for gliomagenesis.

**Conclusion:**

PSA-NCAM represents a valuable biomarker for the prognosis of GBM patients.

## Background

Progress in GBM treatment has been limited. The disease is still characterized by a mortality rate approaching 100% and a lifespan of a few months from point of diagnosis. One of the main impediments to long-term survival is recurrence despite resection of the primary tumor. Recently, radiotherapy associated to adjuvant Temozolomide chemotherapy has increased median survival to 14.2 months [1]. However, the development of more efficient drugs as well as the use of more appropriate therapeutic protocols requires an improved knowledge of the disease, at the molecular level. Molecular diagnostics have emerged as a powerful tool to discover new genes and therapeutic targets, and have realized the proof of principle that personalized medicine can increase survival and cure cancer patients [2]. Various genetic alterations have been identified in GBM, which lead to altered signaling in core pathways [The cancer Genome Atlas Research network: [3]]. However, to date, the main significant prognosis factors evaluated in large cohorts of patients are O6-methylguanine-DNA methyl transferase promoter methylation status, age, extent of resection, performance status, and mini mental state examination [4].

Another focus of intense investigation has been the existence and the role of cancer initiating cells (CIC) in GBM [reviewed in [5]]. Although early studies have focused on CD133 antigen as the CIC marker in GBM [6], more recent studies have pointed out to the existence of CIC characterized by other cell surface markers such as L1CAM [7] or the ganglioside A2B5 [8,9]. In addition, some studies have shown the critical role of olig2, a transcription factor expressed by normal oligodendrocytes. We have reported that olig2 is expressed by all human gliomas whatever their subtype and grade [10], but its level is dramatically increased in highly proliferative, tumorigenic GBM cell lines [11]. Furthermore, olig2 seems to be required for gliomagenesis and proliferation of neural progenitors [12].

We have postulated that PSA-NCAM could be a powerful biomarker for human GBM. In fact, the spatio-temporal pattern of expression of PSA-NCAM, a critical parameter for proper neural morphogenesis, and its biological functions, are consistent with a possible role in GBM. Indeed, polysialylation of NCAM is a broad feature of growing CNS tissue in development, and it is a marker specifically associated with proliferative and plasticity events in adulthood [13,14]. PSA is a unique polymer of α 2,8-N-acetyl neuraminic acid residues added to the fifth Immunoglobulin domain of the membrane bound NCAM. Importantly, by enabling limited cell-cell and cell-extracellular matrix interactions [15], one hallmark of PSA-NCAM is to favor cell migration, a critical feature of the highly invasive behavior of GBM cells.

In order to investigate the expression and the putative role of PSA-NCAM in GBM, we first set up a sensitive ELISA test, which allowed specific detection above 10 pg PSA-NCAM/μg of protein. Results of the ELISA test were correlated with immunohistochemical detection of PSA-NCAM. We studied the expression of PSA-NCAM in a series of 56 GBM samples and found PSA-NCAM expression in 70% of them. Moreover, we report that detectable PSA-NCAM by ELISA test (> 10 pg PSA-NCAM/μg of protein) is an adverse factor of prognosis associated with shorter OS and DFS. We then analyzed PSA-NCAM expression in a human GBM cell line GBM9, previously developed in our laboratory from a patient biopsy and characterized by a highly proliferative behavior and long term sphere formation [9]. We could show that removing PSA-NCAM from these cells down-regulated olig2. Conversely, PSA-NCAM forced expression in the C6 glioma cell line induced olig2 synthesis.

## Methods

### Human subjects and clinical parameters

Surgically resected samples were obtained from 56 adult patients (age>18 years) from the Departments of Neurooncology and Neurosurgery at the Marseille Hospitals (AP-HM), in France from 1986 to 2005. All had primary GBM, histologically proven according to the WHO classification, formalin fixed paraffin embedded tissue. The frozen specimens were stored in the AP-HM tumor bank (Authorization number 2008/70) with written informed consent for tumor banking and study.

All patients benefited from surgery followed by radiotherapy and alkylating agents chemotherapy (nitrosourea), and included long term survivors with a clinical follow up of 8 years. Because the patients were treated before 2005, none of them received the radiotherapy associated to Temozolomide chemotherapy protocol described by Stupp et al. [1].

For all patients, 26 males and 30 females, available clinico-pathological data were the age, preoperative Karnofsky performance status (KPS), extent of surgical removal, OS and DFS. Mean age at diagnosis was 60.4 +/- 10.7, preoperative KPS was less than 70 in 21 patients and greater than 70 in 35 patients. Total surgical excision based on operative chart protocol was achieved in 39 patients and partial excision in 17 patients. The median OS was 12.5 months [mean confidence interval (CI): 9.7-15.4]. Of the 56 patients, 48 had relapse. The median DFS was 6.7 months (mean CI: 4.2-9.2).

In addition, frozen specimens of 3 medulloblastomas (MB) known to highly express PSA-NCAM [16], and non tumorigenic adult brain tissues taken from a patient biopsy in an area containing only non tumoral tissue were used as controls. For PSA-NCAM immunohistochemistry and ELISA analysis, cryostat sections from each patient were checked and consisted of at least 60% tumoral tissue and less than 40% necrotic or non tumoral tissue.

### PSA-NCAM immunohistochemistry

Anti-PSA-NCAM antibody (clone Men B2-2B dilution 1/500, Abcys, Paris, France) was applied on 5 μm cryostat sections available for all GBM and control cases. Immunohistochemistry was performed on a Benchmark XT (Ventana, Tucson, AZ, USA). Controls included omission of primary antibody and abolition of the immunostaining after endoneuraminidase N (endo N, 1/1000; Abcys) treatment, which specifically cleaves alpha 2,8 linked acetyl neuraminic acid residues added to N glycosylation sites on the fifth immunoglobulin domain of NCAM [17]. Immunostaining was analyzed by using a semi-quantitative score. Briefly, the percentage of positive tumor cells per slide (0% to 100%) was multiplied by the main intensity of staining (0: negative; 1: very weak; 2: weak; 3: moderate; 4: intense). PSA-NCAM expression score ranged from 0 to 320 in GBM. It was up to 360 in MB.

### Preparation of tissue and cell extracts, and protein measurement for ELISA

Cells or tissues were homogenized in PBS, 0.1% TritonX-100 and sonicated. The nuclear and mitochondrial fractions and debris were removed by centrifugation at 10000 g, 4°C, for 15 minutes. Total proteins were measured from the supernatant using the BCA kit (Perbio, Brebieres, France). Samples were initially diluted in phosphate buffered saline (PBS) at 37 μg/ml of protein and tested in the ELISA assay. If necessary, the dilution was adjusted to be in the linear range of the standard curve, and the PSA-NCAM concentration calculated in pg PSA-NCAM/μg of total protein.

### Purification of PSA-NCAM for ELISA standard curve, and of NCAM for anti-NCAM antibody generation

PSA-NCAM was purified from postnatal stage P0 mice by affinity chromatography using the previously characterized H28 monoclonal antibody [18] immobilized on an Aminolink column (Perbio) at 10 mg/ml of resin, according to the manufacturer's procedure. Protein concentration was measured using the BCA kit.

NCAM was purified from adult mice brain on the same affinity column, and used to produce a goat polyclonal anti-NCAM antibody (Eurogentec, Orleans, France). The anti-NCAM recognizes the 3 main NCAM isoforms (data not shown).

### PSA-NCAM ELISA

Ninety six-well microtiter plates (Nunc Maxisorp, VWR, Strasbourg, France) were coated with 100 μl of mouse monoclonal antibody directed against PSA-NCAM (1/4000 in PBS) (Abcys). After PSA-NCAM capture, the wells were washed 3 times with PBS (Sigma, Lyon, France) and blocked with 200 μl of PBS containing 5% bovine serum albumin (BSA; Sigma) for 2 h at 37°C. Hundred μl of samples of unknown PSA-NCAM concentration, and standards were then incubated overnight at 4°C. Standards were prepared from known amounts of PSA-NCAM purified from postnatal P0 mice as described above. The dose range for PSA-NCAM standard curve was 0.25 to 16 ng/ml. After 5 washes with PBS containing 0.1% Tween20 (PBST), wells were incubated with 100 μl of goat anti N-CAM prepared from purified mouse NCAM as described above (1/4000; Eurogentec) in PBST, 5% BSA (PBSTB) for 2 h at 37°C. After 5 washes with PBST, wells were incubated with 100 μl of donkey horseradish peroxidase (HRP)-coupled anti-goat antibody (Jackson Laboratory, Ban Harbor, MA) diluted 4000 times in PBSTB for 2 h at 37°C. Plates were washed 5 times with PBST and incubated with 100 μl of Tetramethylbenzidine (TMB; Sigma). TMB tablets were diluted in 0.2 M dibasic sodium phosphate, 0.1 M citric acid, 0.6% H_2_O_2_. The reaction was stopped with 100 μl 1.8 N H_2_SO_4_. Absorbance was read at 450 nm using a 96-well plate reader (Berthold, Thoiry, France) and the concentration of PSA-NCAM in samples was deduced from the standard curve.

For ELISA validation, the rhabdomyosarcoma TE671 cell line was used and grown as previously described [17]. The specificity of the ELISA test was established using endoN. A TE671 extract was digested with endoN for 4 h at 37°C. Non digested and digested extracts were serially diluted and tested in parallel in the same ELISA test to ensure that the signal detected with the ELISA was indeed specific for PSA-NCAM.

### Cell culture, endoneuraminidase treatment and immunocytochemistry

The rat C6 glioma cell line (ATCC-LGC, Molsheim, France) was grown in Dulbecco's modified Eagle's medium (DMEM) supplemented with 10% fetal calf serum (FCS), penicillin (50 Units/ml) and streptomycin (100 μg/ml). The C6-PSA-NCAM cell line (generous gift of M. Fukuda, The Burnham Institute for Medical Research, La Jolla, CA) over expresses PSA-NCAM after transfection with a polysialyltransferase construct (ST8-SiaIV/PST) and has been previously described [19]. C6-PSA-NCAM cells were grown in DMEM supplemented with 10% FCS, penicillin, streptomycin and geneticin (0.5 mg/ml). Both cell lines display similar proliferation rates [20]. For olig2 quantification, 10 mm diameter plates of C6 and C6-PSA-NCAM were used when cells had reached confluence.

The GBM9 cell line was described in our previous study [9]. Cells were grown as neurospheres in Neurobasal medium supplemented with B27, GlutaMAX™, EGF (20 ng/ml) and bFGF (20 ng/ml), both from Peprotech (London, UK). For splitting cells, spheres were spun down (300 g, 5 minutes, RT) and dissociated with 1 to 2 ml Accumax (Abcys, Paris, France) for 30 minutes at 37°C. Single cells suspensions were obtained by mechanical dissociation. Cells were subsequently spun down and resuspended in growth medium. For differentiation experiments, cells were plated in Poly-D-lysine (10 μg/ml) coated multiwell-24 plates (30000 cells/well). All cells were grown in humidified atmosphere containing 5% CO_2 _at 37°C. All tissue culture reagents were from Invitrogen (Cergy Pontoise, Fance).

For studying the effect of PSA-NCAM on differentiation, cells were treated with endoN (1/1000) for 3 days *in vitro*. GBM9 cells were fixed with 4% paraformaldehyde for 15 minutes at room temperature (RT), and washed with PBS. Cells were permeabilized with 0.1% Triton X-100 and 3% BSA for 45 minutes. Primary antibodies were incubated for 2 h at RT in PBS. Following 3 washes of 10 minutes each, appropriate secondary antibodies were incubated for 1 h at RT. Nuclei were stained with DAPI (0.5 μg/ml) for 5 minutes and washed with PBS. Immunofluorescence staining was observed using the Carl Zeiss Observer D1 inverted microscope (Carl Zeiss, Gottigen, Germany) equipped with a 20 × objective. The following antibodies were used: olig2 (rabbit IgG, 1/1000; Abcys), GFAP (mouse IgG, 1/1000; Millipore, Molsheim, France), β3-tubulin (mouse IgG, 1/1000; Sigma), Nestin (mouse IgG, 1/500; Abcys), PSA-NCAM (mouse IgM, 1/1000; Abcys), Alexa555-anti-mouse IgG, CY3-anti-mouse IgM, Alexa488-anti-rabbit IgG (all 1/500; Jackson Laboratory).

### Western Blot

C6 and C6-PSA-NCAM total cell extracts were prepared by scraping cells in 500 μl of 10 mM TrisHCl pH 7.4 containing 0.1% TritonX-100 and Complete™ protease inhibitors (Roche, Mannheim, Germany) followed by sonication. Proteins were run on an 8% SDS-polyacrylamide gel. Western blot was performed according to standard protocols with anti-olig2 (1/4000), anti-PSA-NCAM (1/1000), and anti-α-tubulin (mouse IgG, 1/2000, Sigma). Secondary antibodies were coupled to HRP (1/20000; Jackson Laboratory).

### Statistical analysis

The percentage of olig2 positive cells in GBM9 differentiation assay was analyzed using contingency tables followed by the Fisher's exact test. The Spearman correlation coefficient was used to evaluate the consistency between the 2 methods of PSA-NCAM content measurements in biopsies (immunohistochemistry and ELISA).

For statistics performed on GBM patients, the SPSS 15.0 software for windows (SPSS Inc, Chicago, Illinois, USA) was used. Survival was estimated using the Kaplan-Meier method, and curves were compared using the log-rank test. On multivariate analysis, the effect of potential risk factors including age (< 60 versus ≥ 60), preoperative KPS (< 70 versus ≥ 70), extent of surgical excision (partial versus total), and PSA measurement by ELISA (≤ 10 pg PSA-NCAM/μg of protein versus > 10 pg PSA-NCAM/μg of protein) on the DFS and OS were evaluated using the cox regression proportional hazard models. The threshold for statistical significance was p = 0.05.

## Results

### PSA-NCAM ELISA sensitivity, linearity and specificity

We developed a sandwich ELISA to detect PSA-NCAM by taking advantage of the monoclonal anti-PSA-NCAM developed in our laboratory [21] (Abcys) as the capture antibody. An anti-mouse NCAM was then produced by immunizing goats with NCAM purified from adult mice brain, and used as the detection antibody.

**Figure 1:**
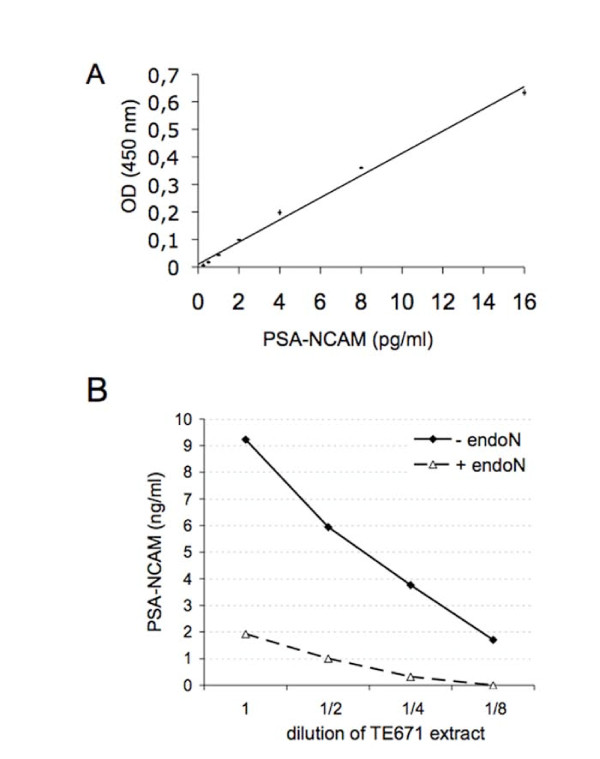
**Standard curve of PSA-NCAM ELISA**. (A) Increasing amounts of purified PSA-NCAM from P0 mouse brains were detected by the developed ELISA. Data are presented as mean +/- standard deviation of duplicate measurements. (B) ELISA test on serial dilutions of TE671 extract expressing PSA-NCAM or treated with endoN.

A linear relationship could be established between known amounts of PSA-NCAM and optical density readings between 0.25 and 16 ng PSA-NCAM/ml (Figure 1A). Tissue extracts were tested after dilution at 37 μg of protein/ml; therefore the corresponding limit of detection in such sample is 6.75 pg PSA-NCAM/μg of protein. The test was linear over 2 logs around the concentration tested. Intra-assay coefficient of variation was less than 5% (n = 3). Although the ELISA was designed with appropriate antibodies to detect PSA-NCAM, we first verified the specificity of the ELISA by digesting PSA-NCAM from an extract of TE 671 cells known to express PSA-NCAM [17]. After verifying that endoN indeed digested PSA-NCAM by Western blot (not shown), a serial dilution of TE671 extract treated or not with endoN was prepared and tested by ELISA. Data shown on Figure 1B indicate that the signal obtained in absence of endoN is specific for PSA-NCAM as it was completely abolished at most dilutions of the extract after enzymatic digestion. In these diluted extracts, PSA-NCAM was more concentrated than in the GBM biopsies, ensuring that the signal measured in GBM biopsies was entirely PSA-NCAM specific. However, a limitation of our test is that although the anti-PSA antibody allows to reveal PSA, it does not discriminate between different lengths of PSA chains. In order to accurately assess PSA-NCAM level from GBM biopsies, its expression in normal brain tissue had to be determined. Adult human brain cortex expresses PSA-NCAM only on rare cells [22]. In non tumoral adult human brain tissue, a signal equivalent to 8 pg PSA-NCAM/μg of protein was measured, and PSA-NCAM was below detection in adult mouse cortical tissues (n = 4). Moreover, the inter assay variability for these samples was of 10 pg/μg of protein. Therefore, the threshold of significance for specific detection of PSA-NCAM was set to 10 pg PSA-NCAM/μg of protein. This value was chosen to classify groups of GBM patients that express either less or more than 10 pg PSA-NCAM/μg of protein.

**Figure 2:**
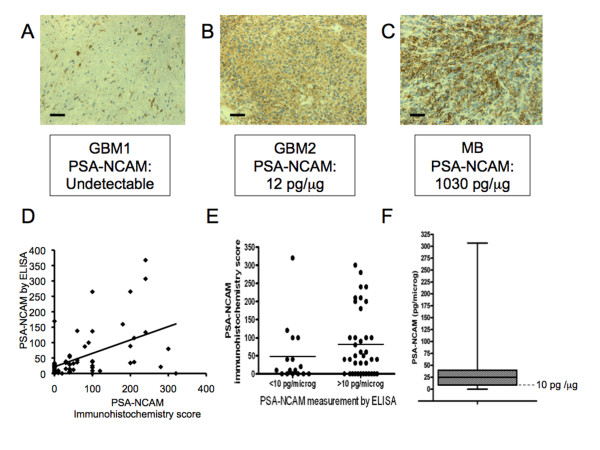
**PSA-NCAM measurements by immunohistochemistry and ELISA, and distribution of PSA-NCAM measurement by ELISA in 56 GBM biopsies**. PSA-NCAM immunohistochemistry on two GBM biopsies with below detection (A) or positive (B) staining, and on a MB biopsy highly expressing PSA-NCAM (C); scale bar: 33 μm. (D) Individual measurements of PSA-NCAM in the 56 GBM, by ELISA and Immunohistochemistry. (E) Individual measurements of PSA-NCAM by ELISA and Immunohistochemistry in two groups (PSA-NCAM<10 pg/μg of protein and PSA-NCAM>10 pg/μg of protein). Lines indicate the median in each group (F) Distribution of PSA-NCAM content from 56 biopsies, represented as boxplot with first quartile (9 pg PSA-NCAM/μg of protein), median (25 pg PSA-NCAM/μg of protein) and third quartile (40 pg PSA-NCAM/μg of protein). The dotted line corresponds to 10 pg PSA-NCAM/μg of protein (level of sensitivity of PSA-NCAM detection in GBM tissue).

Embryonic human brain extract served as positive control since it is known to express high level of PSA-NCAM, as do medulloblastoma (MB) tissues [16], which was confirmed in the current study by immunohistochemistry (Figure 2C) and ELISA for MB (150-1030 pg PSA-NCAM/μg of protein, n = 3) and embryonic human brain (1600 pg PSA-NCAM/μg of protein).

### PSA-NCAM measurement by ELISA is correlated to immunohistochemistry analysis

Immunohistochemistry has been so far the standard method in anatomopathology laboratories, including using the PSA-NCAM antibody [16,23]. Therefore, we verified that the results obtained using the ELISA test correlated with the immunohistochemistry results. Figure 2A shows biopsies with various degrees of PSA-NCAM immunohistochemistry staining with the corresponding ELISA measurements. They illustrate weak or medium PSA-NCAM immunostaining in two GBM samples, which generated a corresponding PSA-NCAM value by ELISA, similar to non-tumoral adult human brain tissue for GBM1 (Figure 2A) and higher for GBM2 (Figure 2B). It is worth noticing that PSA-NCAM expression was different from one GBM to another: either weak and diffuse in large area of the biopsy in some cases (Figure 2B), or strong but restricted to fewer tumor cells in others (Figure 2A), this last pattern of expression being more frequent. We also included in our study 3 MB known to express high levels of PSA [16]. These MB samples were highly stained by immunohistochemistry (score 360 to 400) (Figure 2C) and displayed high PSA-NCAM concentrations by ELISA (150-1030 pg PSA-NCAM/μg of protein, n = 3). In total, a series of 56 GBM samples were analyzed by immunohistochemistry and given a score, and by ELISA. Figure 2D shows the 56 individual values of PSA-NCAM measured by ELISA and immunohistochemistry, and on Figure 2E, patients were separated in two groups (ELISA PSA-NCAM <10 pg/μg of protein and PSA-NCAM >10 pg/μg of protein). Spearman correlation test shows a statistical positive correlation between the two methods of PSA-NCAM measurement (Rho = 0.439, p = 0.005).

### PSA-NCAM is expressed in 70% of 56 GBM biopsies

We quantified PSA from 56 GBM biopsies. As much as 70% of them expressed PSA-NCAM at levels above 10 pg PSA-NCAM/μg of protein. The median was of 24.73 pg PSA-NCAM/μg of protein. Apart from some very high values as depicted on the histogram (Figure 2F), 50% of the biopsies gathered around the median. It should be noted that 90% of them contained less PSA-NCAM than the MB with the lowest PSA-NCAM content (150 pg PSA-NCAM/μg of protein).

### PSA-NCAM expression in GBM is correlated to shorter OS and DFS

**Figure 3:**
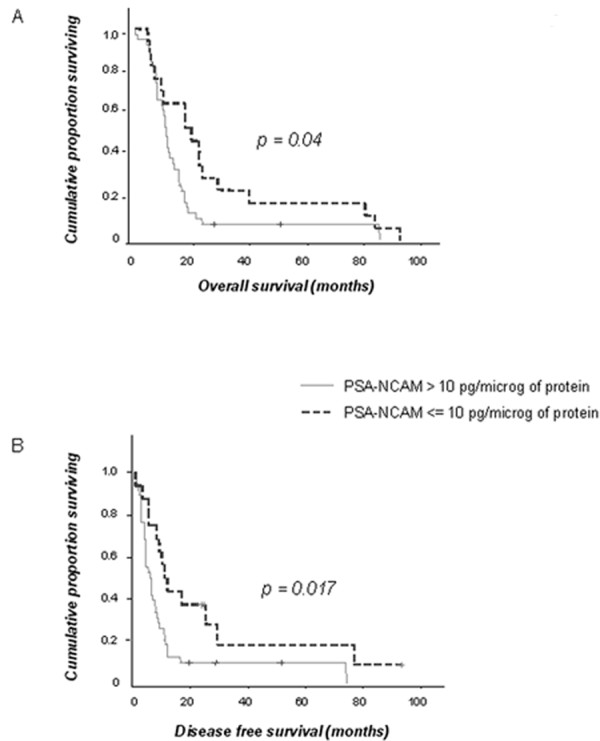
**PSA-NCAM is negatively correlated to overall (OS) and disease free (DFS) survival**. Survival curves of patients from the two groups: PSA-NCAM>10 pg/μg of protein (n = 39 for OS; n = 38 for DFS) and PSA/NCAM ≤ 10 pg/μg of protein (n = 17 for OS; n = 16 for DFS). (A) OS: Kaplan-Meier curves indicate for each time point the proportion of patients still alive at that time. (B) DFS: Kaplan-Meier curves indicate for each time point the proportion of patients that did not yet have a relapse at that time. (+): censored patients.

In this series, none of the clinical factors including age, preoperative KPS and extent of surgical removal was correlated to OS or DFS. The patient cohort includes long-term survivors, and surgical resection was not systematically analyzed by MRI. These factors could have prevented to reveal the prognosis value of these classical clinical parameters. However, PSA-NCAM expression was significantly associated to OS [median OS: 20.8 months (CI:14.2-27.4) in patients with PSA-NCAM GBM content ≤ 10 pg/μg of protein versus 12.2 months (CI:11.0-13.5) in the others; p = 0.04, n = 56] (Figure 3A). PSA-NCAM expression was also statistically correlated to DFS (median DFS: 11.2 months (CI:7.6-14.8) in patients with PSA-NCAM GBM content ≤ 10 pg/μg of protein versus 6 months (CI:3.4-8.5) in the others; p = 0.017, n = 54] (Figure 3B).

On multivariate analysis (age, KPS and extent of surgical excision), PSA-NCAM expression was the unique predictive parameter of OS [hazard ratio (HR): 2.01 for patients suffering from a GBM with PSA-NCAM content >10 pg/μg of protein (CI:1.01-4); p = 0.046, n = 56] and DFS [HR: 2.89 for patients suffering from a GBM with PSA-NCAM content >10 pg/μg of protein (CI:1.34-6.27); p = 0.007, n = 54].

### PSA-NCAM regulates olig2 expression in glioma

**Figure 4:**
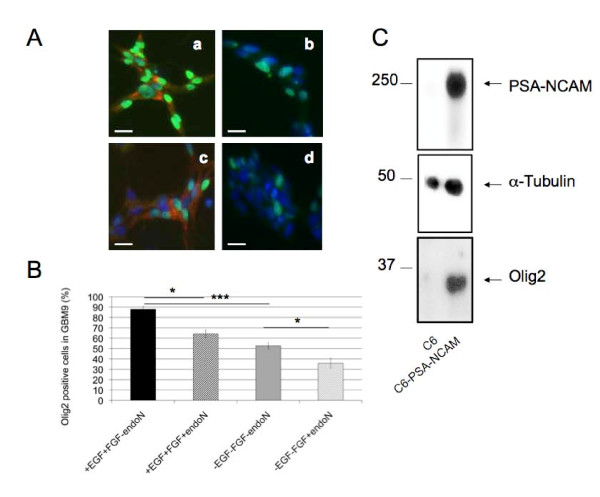
**Olig2 is positively correlated to PSA-NCAM**. (A) Immunostaining of GBM9 cells with anti-olig2 (green, Alexa488) and anti-PSA-NCAM (red, CY3) in the presence of EGF + FGF (a), EGF + FGF + endoN (b), no growth factor (c), endoN and no growth factor (d). Cells were counterstained with DAPI (blue); scale bar: 20 μm. (B) Quantification of immunostaining described above from a representative experiment out of 2 performed. Percentage of olig2^+ ^cells is presented as mean +/- standard deviation of the percentage of olig2^+ ^cells grown in presence of EGF and FGF, from at least 5 independent fields of view per condition. Between 174 and 571 cells per condition were counted. (*: p < 0.05, ***: p < 0.001; Fisher's test). (C) Representative western blot out of 3 independent cultures of C6 and C6-PSA-NCAM with anti-PSA-NCAM, anti-α-tubulin and anti-olig2 antibodies.

To further elucidate the role that PSA-NCAM could play in GB, we investigated whether PSA removal using endoN affected the properties of the GBM9 cell line we previously developed and characterized as a highly proliferative neurosphere forming cell line [9]. To this end we compared features of endoN treated and non treated cells in different culture conditions. In basal conditions, cells were grown in presence of EGF and FGF [9]. In a representative experiment described on Figure 4, out of two performed, 88% of the GBM9 cells expressed the olig2 transcription factor. Representative pictures taken from at least 5 independent fields are shown on Figure 4A. Under conditions of PSA removal, the percentage of olig2 positive cells decreased to 64% (Figure 4B). If EGF and FGF were removed from the culture medium, 52% of the cells were olig2 positive, and this percentage further decreased to 35% after PSA digestion. The mean of at least 5 fields is represented on Figure 4B. In the two experiments performed, the percentage of cells losing olig2 was 30 and 46% in the presence of EGF and FGF, and 17 and 35% in the absence of growth factors, respectively. PSA removal was capable of decreasing significantly olig2 content in this GB cell line both under proliferative conditions in the presence of EGF and FGF (p = 0.0153 and p = 0.0399 for the 2 experiments, respectively), and in their absence (p = 0.0289 and p = 0.0297 for the 2 experiments, respectively). We found no effect of PSA removal on the percentage of other cell lineages generated (GFAP^+ ^astrocytes, β3-tubulin^+ ^neurons or Nestin^+ ^progenitors) neither in presence, nor in absence of growth factors (not shown).

To uncover a possible causal relationship between olig2 and PSA expression, we tested whether PSA-NCAM gain of function would affect olig2 expression. We chose C6 cells which displayed undetectable PSA-NCAM by ELISA or Western blot and show many features of GB tumors after intracranial injection such as an undifferentiated morphology [20], neovascularization [24], diffuse infiltrating borders and invasion of the surrounding tissue by isolated cells [25]. Moreover, PSA-NCAM forced expression in these cells has been shown to be associated with their increased migration when transplanted in mice [20]. Therefore, we compared olig2 content in PSA-NCAM negative C6, and C6-PSA-NCAM that contained 19.5 pg PSA-NCAM/μg of protein as measured by ELISA. Whereas C6 cells did not express olig2, C6-PSA-NCAM displayed strong olig2 expression indicating that in these cells, forced expression of PSA is sufficient to induce olig2 as shown by western blot analysis in Figure 4C.

## Discussion

In the current study, we show that PSA-NCAM is a prognosis factor in GBM, by using a new ELISA assay for PSA-NCAM. This test allows quantifying accurately, specifically, and with high sensitivity PSA-NCAM on small amounts of biopsy materials. The results obtained were well correlated to immunohistochemistry analysis, showing the robustness of the assay. However, some samples displayed different levels of PSA, depending on the method of measurement. The non-linear scoring by IHC and the possible heterogeneity of the tumor may partly explain the discrepancy between the two methods. High PSA values by ELISA could therefore represent false positives (type I error) as their IHC score was 0 in the PSA_ELISA _>10 pg/μg of protein group (12 cases), or false negatives (type II error) as their IHC value was much higher than the median IHC score in the PSA_ELISA _<10 pg/μg of protein group (4 cases). Nevertheless, when confronted with the outcome of patients, the ELISA measurement of these samples seemed to be more predictive. Three out of the 4 samples that had low PSA by ELISA came from patients that had an overall survival > 20.8 months (median OS in the PSA_ELISA _<10 pg/μg of protein group) and disease free survival > 11.2 months (median DFS in the PSA_ELISA _<10 pg/μg of protein group). Nine out of the 12 samples that had high PSA by ELISA came from patients that had an overall survival < 12.2 months (median OS in the PSA_ELISA _>10 pg/μg of protein group) and disease free survival <6 months (median DFS in the PSA_ELISA _>10 pg/μg of protein group).

Importantly, this test presents a number of advantages over currently in use measurement techniques, immunohistochemical detection and Western blot, which are semi-quantitative. Therefore, once validated on a larger scale, it fulfills the criteria (quantitative test, sensitivity and specificity) that would allow its use in the clinic for GBM and other cancers. Indeed, in addition to the prognosis value of PSA-NCAM in GBM demonstrated here, evidence from our and others' studies have shown that PSA-NCAM is also a reliable marker in many types of cancers of the CNS and other organs, and that it is involved in the process of metastasis [16,23,26-28]. A correlation could also be established between the level of PSA-NCAM and the severity of the disease and/or the response to treatment and relapse in a large variety of cancer including medulloblastoma [28], neuroblastoma [29], rhabdomyosarcoma [30] and small cell lung carcinoma [31]. PSA-NCAM in non-small cell lung cancer biopsies was correlated with tumor progression and was an independent prognosis factor [32]. NCAM expression has also been evaluated in glioma [20], and other tumor types [31], but NCAM immunoreactivity does not differentiate polysialylated from non polysialylated NCAM. Interpretations of NCAM expression results have therefore been limited due to the omitted consideration of NCAM polysialylation, post-translational modification that affects dramatically NCAM binding properties and intermembrane repulsion [15].

In gliomas, PSA-NCAM has been detected in GBM explants [33] and on biopsies more frequently in diffuse astrocytoma cells, which spread extensively [20]. It has also been shown to confer invasive properties to glioma cells in animal models [20]. However, although some studies have reported biomarkers of different grades of glioma [34,35], investigations showing the prognosis value of molecular factors by multivariate analysis, specifically in the GBM subgroup, have been limited, and most used semi-quantitative methods or mRNA analysis [36,37]. Therefore, the methodology developed here and the finding of PSA-NCAM as a prognosis marker in GBM appears significant.

We found that 70% of biopsies expressed PSA-NCAM at levels above the level set up as background, with some variability. Moreover, GBM showed considerably lower levels than MB. A likely explanation for this difference is that MB cells are all in the same differentiation state, engaged in a neuronal phenotype, whereas in GBM, cells from very immature to differentiated phenotype can be observed (personal observations). The contrasted low PSA levels in GBM versus high levels in MB could also explain the highly metastatic versus non metastatic behavior of MB versus GBM, respectively.

The content of PSA-NCAM in GBM biopsies might result from at least two phenomena: the number of cells expressing PSA-NCAM and the amount of PSA-NCAM expressed by individual cells. The different patterns of PSA-NCAM expression observed by immunohistochemistry in GBM are in keeping with these hypotheses as some show a rather low level on many cells spread over the tissue whereas for others high expression is exhibited only by islets of cells. However, since the tumor is not strictly homogeneous, the different staining patterns that can be observed cannot be assumed to be respresentative of the entire tumor of a given patient. Cells expressing PSA-NCAM could be either progenitors and/or cells exhibiting an altered differentiation process due to transformation, although we cannot exclude that in some cases PSA-NCAM positive cells are cells from the subventricular zone attracted by the tumor [38]. PSA expression is under transcriptional control, a mechanism that can be perturbed following oncogenic transformation, and the enzymes polysialyltransferases that transfer PSA to NCAM represent obvious targets. In addition, several post-translational mechanisms that have been shown to regulate the expression dynamics could be at play [39,40].

Despite variations of expression, PSA-NCAM expression was correlated with poor prognosis for both OS and DFS. Although at this stage whether PSA-NCAM molecule itself plays a causal role or is a downstream consequence of changes in tumorigenesis is not known, some hypotheses may be formulated to explain a negative role of PSA-NCAM in survival. First, PSA-NCAM may be carried by undifferentiated cells in the tumor capable of cancer initiation and resistance to therapy [7]. Indeed, cumulative studies suggest that persistence of PSA-NCAM could prevent differentiation. During development, a defined spatial and temporal window of expression of PSA-NCAM illustrates that this molecule constitutes a very effective cue when plasticity and precise timing of differentiation is required [40]. A prominent role of PSA in differentiation is also supported by its capacity to delay oligodendrocyte maturation events such as differentiation and myelinating potential [19,41,42] or neuronal differentiation [43,44]. Moreover, PSA-NCAM is an attractive candidate to be expressed by CIC since it is characterized by a strong and ubiquitous expression during embryonic development in multipotent or lineage restricted precursors, and a stem cell niche marker in the adult CNS, mainly in the SVZ and hippocampus producing neuroblasts throughout life. Polysialylation could therefore support the maintenance of an immature phenotype. Transplantation studies with PSA^+ ^and PSA^- ^GBM cell populations will be required to answer whether PSA-NCAM defines cell populations with CIC properties.

Second, PSA-NCAM could favor infiltration of cells in the surrounding healthy tissue and be responsible for earlier relapse, which may subsequently lead to decreased OS as disseminated tumor cells play a critical role in GBM recurrence. This PSA-NCAM enhancing effect on migration was indeed demonstrated in healthy tissue and in disease models such as rhabdomyosarcoma metastasis mouse model [27] and after grafting of PSA-expressing C6 glioma cells in mouse brain [20].

Third, PSA-NCAM might trigger proliferation and enhance GBM growth. This could occur via an increased sensitivity of PSA-NCAM positive cells to growth factors such as PDGF, as shown for oligodendrocytes [45], or GDNF [17]. Interestingly PDGF receptor expression was required for oligodendrocytes to generate gliomas [46]. An alternative could be the prevention of contact mediated inhibition of proliferation as exerted in astrocytes [47] or neuroblastoma cell lines [48].

Importantly, we also found olig2 expression to be regulated by PSA-NCAM expression in the GBM9 cell line or to be activated in C6 after PSA-NCAM expression. The mechanisms underlying olig2 regulation could be related to PSA-NCAM mediated alterations of cell proliferation, survival or differentiation [19,41-44,48] and remain to be elucidated. Olig2 has been described as a negative regulator of astrocytic differentiation pathway [49], to be required for gliomagenesis and to be coupled to an inhibition of the antiproliferative p21 pathway [12]. Olig2 is also known to play an important role in proliferation and fate determination in the development of the nervous system and adulthood [50]. Therefore, one of the consequences of PSA-NCAM up regulation in GBM could be olig2 increase and its downstream growth and differentiation consequences. Further investigations of the impact of olig2 levels in relation to GBM patients' survival appear compelling.

In conclusion, PSA-NCAM is another example of molecules having a dual role, during development as fundamental regulators of pattern formation and differentiation, and in cancer. Moreover, the role of an adhesion molecule of the immunoglobulin family in cancer stem cell is not unprecedented since L1CAM was recently shown to be required to suppress glioma growth of CD133^+ ^cells *in vitro *and *in vivo *[7]. These findings constitute the first evidence that PSA-NCAM expression correlates with GBM patient survival. This novel biomarker for GBM may represent an interesting molecular tool for diagnosis and prognosis in clinical use. This may also have important clinical implications for the treatment of GBM patients as previously suggested with chemicals that alter PSA expression with the goal to modulate polysialylation in tumors [51]. This study warrants further investigation of PSA-NCAM with regard to response to treatment in future prospective studies on a larger number of GBM patients.

## Conclusions

We present PSA-NCAM as a new candidate prognosis biomarker for GBM. It represents immediate valuable information to improve life conditions of patients and clinical decisions, and represents a prospective therapeutic target and stratifying factor for clinical trials. This novel finding, next to the negative impact of PSA-NCAM on survival and metastasis in other cancers, emphasizes a critical and general role of PSA-NCAM in cancer evolution. The new ELISA test described in the current study was characterized as an accurate and specific quantitative mean to measure PSA-NCAM from a small amount of biopsy, and therefore is a powerful tool, notably for validation in a larger patient cohort, but also to investigate PSA-NCAM with regard to response to treatment. In addition, this new ELISA test can be applied to all tumors expressing PSA-NCAM.

## Competing interests

M-C. Amoureux receives her salary from Abcys SA. Study design, analysis and manuscript writing was entirely overseen by IBDML and Hôpital de la Timone.

## Authors' contributions

MCA performed the PSA-NCAM ELISA measurements, the olig2/PSA-NCAM immunocytochemistry and Western blot experiments, designed the *in vitro *experiments, performed the analysis of the data and wrote the manuscript. GR made substantial intellectual contribution to the study and the drafting of the manuscript. BC performed the PSA-NCAM immmunohistochemistry and scoring. AL performed the statistical analysis. PM and OC recruited the patients in a non-selective, consecutive manner, established the diagnosis, performed the surgery and defined the inclusion criteria for the study. DFB participated in study conception, design and establishment of the patients' diagnosis, interpretation of data and drafting of the manuscript. All authors read and approved the manuscript.

## Pre-publication history

The pre-publication history for this paper can be accessed here:

http://www.biomedcentral.com/1471-2407/10/91/prepub
